# Investigating the antioxidant, antibacterial, and antifungal properties of *typha domingensis* leaves assisted synthesized zinc oxide nanoparticles

**DOI:** 10.1371/journal.pone.0337942

**Published:** 2026-01-13

**Authors:** Md. Suzon Ahmed, Md. Ariful Islam Setu, Atasi Goswami, Mst. Sabiha Sultana, Palash Kumar Dhar, Md. Rezaul Haque, Sagar Kumar Dutta

**Affiliations:** 1 Chemistry Discipline, Khulna University, Khulna, Bangladesh; 2 Agrotechnology Discipline, Khulna University, Khulna, Bangladesh; National Chung Cheng University, Taiwan & Australian Center for Sustainable Development Research and Innovation (ACSDRI), AUSTRALIA

## Abstract

Green-synthesized nanoparticles (NPs) offer unique properties over traditional approaches, with applications in coatings, catalysts, packaging, environmental cleaning, and medicine. This research involves the synthesis of Zinc oxide nanoparticles (ZnO NPs) utilizing aqueous leaf extract of *Typha domingensis* (locally known as Hogla pata) as a reducing agent. The biosynthesized NPs were then characterized using ultraviolet-visible spectroscopy (UV-Vis), Fourier transform infrared (FTIR) spectroscopy, field emission scanning electron microscopy (FESEM), and energy-dispersive X-ray (EDX) spectroscopy. The XRD result revealed that the synthesized NPs were enriched with (101) facets, which were similar to the standard JCPDS card no 00-036-1451. The size of the ZnO NPs was determined from XRD data utilizing the Debye-Scherrer equation and found to be around 26.74 nm. Moreover, the SEM analysis showed that the shape of the NPs was agglomerated spherical. The 2,2-diphenyl-1-picrylhydrazyl (DPPH) free radical showed dose-dependent antioxidant activity against synthesized ZnO NPs, with an average IC_50_ of 32.25 µg/mL. The antibacterial efficacy of green-synthesized ZnO NPs was assessed against fungal and bacterial pathogens in antifungal experiments. *Colletotrichum sp.* showed the highest sensitivity to ZnO NPs, with a 20 ± 0.80 mm zone of inhibition (ZOI) and 22.22% inhibition, whereas *Fusarium sp.* had a 17 ± 0.68 mm ZOI and 18.89% inhibition at a dosage of 2 mg/mL. ZnO NPs also exhibited antibacterial activity against two gram-negative bacteria, *Coliform sp.* and *Salmonella sp.*, both demonstrating a 12 mm ZOI and 13.33% inhibition at 5.0 mg/mL. These results demonstrate that ZnO NPs biosynthesized with *Typha domingensis* leaf extract exhibit versatile antimicrobial and antioxidant properties, offering a safe and sustainable alternative to synthetic compounds.

## Introduction

Nanotechnology has revolutionized diverse scientific and industrial sectors by enabling the fabrication of materials with dimensions below 100 nanometers, imparting unique physicochemical and biological properties that differ significantly from their bulk counterparts [1–4]. The extraordinary surface-area-to-volume ratio and quantum effects of nanoparticles enhance their reactivity and functional performance, making them invaluable in medicine, agriculture, environmental protection, and biosensing applications [5–7]. Among the wide range of engineered nanomaterials, ZnO NPs are particularly valued for their multifunctional capabilities, including antioxidant, antibacterial, and antifungal activities, photocatalytic properties, alongside their favorable biocompatibility and low toxicity [8–10]. ZnO NPs can neutralize reactive oxygen species (ROS) and inhibit pathogenic bacteria and fungi, positioning them as promising alternatives to conventional antibiotics and fungicides, especially amidst rising antimicrobial resistance [8,9,11]. Traditional methods for producing ZnO NPs, including chemical vapor deposition, sol-gel procedures, and hydrothermal approaches, are well-established, but they frequently use toxic chemicals and energy-intensive processes. This creates serious environmental and safety concerns [12–15]. In response, green synthesis has evolved as an environmentally benign alternative, producing nanoparticles using biological systems such as plants, fungi, and bacteria. These approaches not only reduce toxicity but also encourage sustainable practices that align with the fundamentals of green chemistry by lowering the usage of harmful chemicals and energy consumption [12,16–18].

Among the several plant species appropriate for green nanoparticle synthesis, *Typha domingensis*, a member of the *Typhaceae* family, generally known as cattails, stands out for its important therapeutic and ecological properties. This perennial herbaceous plant is easily recognized by its long green stalks and unique brown, sausage-shaped blooming heads and is primarily found in wetland regions such as marshes, swamps, and shallow water [19,20]. *Typha domingensis* has a long history of use in traditional medicine in various cultures, with a wide range of therapeutic uses such as promoting wound healing, treating burns, and producing diuretic effects. The importance of *Typha* species in traditional healing practices is well-documented due to their medicinal uses. In Turkish folk medicine, the female flowers of *Typha* are used to promote wound healing and stop bleeding. Similarly, in Traditional Chinese Medicine, Pollen *Typhae* is highly valued for its ability to stop bleeding and is employed in the treatment of nosebleeds, hematemesis, hematuria, and uterine haemorrhage. Furthermore, it has been observed that *Typha* pollen has the potential to decrease cholesterol, heal external wounds, and alleviate inflammation, emphasizing its bioactive properties. Recent phytochemical composition investigations suggest that *Typha domingensis* includes a high concentration of bioactive compounds such as alkaloids, flavonoids, tannins, and phenols [19–21]. These compounds play an important role in increasing metal ion reduction and ensuring nanoparticle stability. This not only assists in creating ZnO NPs but also improves their biological characteristics, enabling them to function effectively as antioxidants, antibacterial agents, and antifungal agents. Additionally, the economic benefits of using *Typha domingensis* for nanoparticle production are significant due to its widespread availability and cost-effectiveness in comparison to other plant sources. *Typha domingensis* has a long medicinal history and a rich phytochemical profile, making it a potential candidate for the eco-friendly production of ZnO NPs [21–23].

ZnO NPs are extensively researched for their antioxidant abilities, which can help reduce oxidative stress, a substantial contributor to aging, cancer, and numerous neurological illnesses. Zinc oxide NPs decrease oxidative damage to cells by scavenging free radicals, which helps to support general health and wellness [24,25]. Moreover, ZnO NPs have strong antimicrobial properties and can effectively fight bacterial and fungal pathogens by producing reactive oxygen species that disturb microbial cell membranes, ultimately causing cell death [26,27]. This makes ZnO NPs extremely essential in combating infections, especially considering the increasing threat of antibiotic-resistant pathogens [28,29]. Moreover, in the field of agriculture, ZnO NPs offer a sustainable substitute for traditional chemical pesticides and fungicides, offering a natural remedy for safeguarding crops against pathogenic fungi and bacteria [30–32]. NPs have been added to packaging materials in the food industry to stop the growth of microorganisms and prolong the freshness of perishable products. Such numerous uses illustrate ZnO NPs’ adaptability in a variety of disciplines.

The increasing global concerns over antimicrobial resistance, environmental pollution from synthetic chemical processes, and the need for sustainable nanomaterial production motivate the search for eco-friendly synthesis routes. *Typha domingensis,* a phytochemically rich, widely available wetland plant, offers an untapped opportunity to produce bioactive ZnO NPs without hazardous chemicals or energy-intensive procedures. This study is driven by the dual goal of utilizing an abundant natural resource and producing ZnO NPs with enhanced biological performance. The main objectives are to (1) biosynthesize ZnO NPs using aqueous *Typha domingensis* leaf extract, (2) characterize their physicochemical properties, and (3) evaluate their antioxidant, antibacterial, and antifungal activities for potential applications in healthcare, agriculture, and environmental protection.

Although many plant-based ZnO NP syntheses have been reported, *Typha domingensis* remains largely unexplored. It has a high content of flavonoids, tannins, alkaloids, and phenols that could influence nanoparticle stability, size, and bioactivity. Furthermore, no comprehensive study has yet correlated the plant’s distinctive phytochemical composition with ZnO NP morphology, crystallinity, and multifunctional biological effects. This work addresses these gaps by introducing an underutilized, ecologically abundant plant species as both a reducing and capping agent, enabling the production of multifunctional ZnO NPs through a sustainable green synthesis route. The novelty lies in exploiting *Typha domingensis* not only as a green synthesis medium but also as a natural enhancer of nanoparticle bioactivity, offering a cost-effective and environmentally responsible method for producing ZnO NPs with diverse real-world applications [33,34].

## Experiments

### Chemicals and materials

Merck, Germany, provided the concentrated nitric acid (HNO_3_, 65%), sodium hydroxide (NaOH), ethanol (C_2_H_5_OH), sulfuric acid (H_2_SO_4_, 98%), hydrochloric acid (HCl, 37%), and ferric chloride (FeCl_3_). Sigma-Aldrich provided the zinc nitrate hexahydrate $\left(Zn{\left(NO_{\text{3}}\right)}_{\text{2}}.\text{6}H_{\text{2}}O\right)$, chloroform (CHCl_3_), commercial ZnO NPs and antibiotics. In the lab, Mayer’s reagent (HgCl₂ + KI) was made. Ascorbic acid (C_6_H_8_O_6_) and 2,2-diphenyl-1-picrylhydrazyl (DPPH) are provided by Sigma-Aldrich. Tween 80, 0.9% sodium chloride (NaCl) solution, and dimethyl sulfoxide (DMSO) were also acquired from Sigma-Aldrich. MacConkey Sorbitol Agar (SMAC) and Potato Dextrose Agar (PDA) were acquired from HiMedia Laboratories. The sterile 0.45 µm syringe filters were acquired from Merck Millipore. The chemicals and solvents were used without any further purification. Double-deionized water from RCI Labscan Limited was used continuously throughout the whole investigation.

### Collection of plant materials

Fresh *Typha domingensis* leaves were gathered from Khulna University Campus, located in Khulna District, Bangladesh. Christiaan Hendrik Persoon (1761–1836), a renowned mycologist and botanist, gave the plant its name. It is a member of the *Typhaceae* family; its genus is *Typha* and its species is *domingensis*. It is locally known as Hogla pata, and Professor Dr Mst. Sabiha Sultana, PhD in plant pathology, who has over five years of experience in plant identification at Agrotechnology Discipline of Khulna University, Khulna-9208, Bangladesh, confirms its identity.

### Preparing plant extract

**Fig 1:**
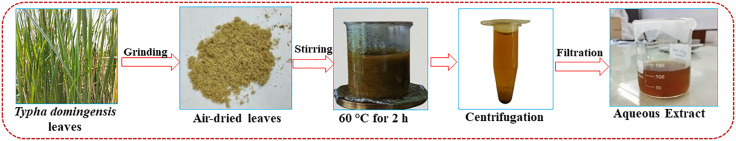
Preparation of plant extract from *Typha domingensis* leaves.

The plant materials were left to dry in a shaded area for 20 days to prevent the degradation of active compounds caused by sunlight exposure. The leaves were dried, then pulverized. In a beaker, a mixture of 20 grams of finely powdered *Typha domingensis* leaves and 220 mL of distilled water was stirred at 500 rpm for 2 hours at 60 °C. After cooling the mixture, it was filtered through filter paper. To eliminate any remaining coarse or insoluble particles, the supernatant was centrifuged at 5000 rpm for 10 minutes and then filtered again. The final extract was then refrigerated at 2 °C for future use [35]. This process is illustrated in Fig 1. The wine-red color of the *Typha domingensis* leaf extract shown in this figure is due to the presence of various phytochemicals, particularly polyphenols, flavonoids, tannins, and other conjugated aromatic compounds, as detected in the phytochemical tests and GC–MS analysis. These compounds have extended π-electron systems that strongly absorb in the visible region, often producing red, brown, or amber colors in aqueous solution.

### Phytochemical testing

The plant extract has been tested for phytochemical compounds, as shown in Table 1.

**Table 1:** Phytochemical analysis of *Typha domingensis* leaf extract.

Phytochemicals	Aqueous Extract
Saponins	**+**
Anthocyanosides	**–**
Tannins	**+**
Steroids	**+**
Phenols	**+**
Alkaloids	**+**
Proteins	**–**
Flavonoids	**+**

1) **Test for Saponins**

2 mL of distilled water were mixed with the plant filtrate in a 1:2 ratio. The mixture was shaken vigorously and then allowed to stand for ten minutes. If foam forms on the mixture’s surface and persists for longer than ten minutes, saponins are present [36].

2) **Test for Anthocyanosides**

To detect anthocyanosides, 1 mL of plant filtrate was mixed with 5 mL of dilute HCl. A pale pink color was seen, which indicates the presence of anthocyanoside [36].

3) **Test for Tannins**

3 mL solution of *Typha domingensis* extract was added to a test tube, followed by 1 mL of 5% ferric chloride solution. Greenish black precipitates indicate the presence of tannins [37].

4) **Test for Steroids**

1 mL of *Typha domingensis* extract was mixed with 5 mL of chloroform, followed by 1 mL of sulfuric acid. A red color indicates the presence of steroids [37].

5) **Test for Phenols**

1 mL of *Typha domingensis* was mixed with 2 mL of distilled water. A solution of 10% ferric chloride was added drop by drop. Phenols are present in the greenish-black precipitate [37].

6) **Test for Alkaloids**

Mayer’s test The Mayer’s test involved mixing 2 mL of *Typha domingensis* extract with 5 mL of diluted 1% hydrochloric acid in a test tube. After that, 1 mL of Mayer’s reagent was added. A white or creamy-white precipitate suggests the presence of alkaloids [37].

7) **Test for Proteins**

When 2 mL of extract were treated with only a few drops of concentrated nitric (HNO_3_) acid, the solution turned yellow, indicating the presence of proteins [38].

8) **Test for Flavonoids**

2 mL of plant extract were taken in a test tube, and then a few drops of 10% NaOH were added; an intense yellow color indicates the formation of flavonoids [38].

### Methodology of GC-MS analysis

Gas chromatography-mass spectrometry analysis was carried out with Clarus^®^690 gas chromatograph (PerkinElmer, CA, USA) using a column (Elite-35, 30 m length, 0.25 mm diameter, 0.25 µm thickness of film) and it was equipped with Clarus^®^ SQ 8 C mass spectrophotometer (PerkinElmer, CA, USA). 1.0 µL sample was injected (spitless mode) and pure Helium (99.999%) was used as a carrier gas at a constant flow rate (1 mL/min) for 40 min run time. The sample was analysed in EI (electron ionization) mode at high energy (70 eV). Though the inlet temperature was constant at 280 °C, column oven temperature was set at 60 °C (for 0 min), raised at 5 °C per minute to 240 °C and held for 4 min [39]. The sample compounds were identified by comparing them to the National Institute of Standards and Technology (NIST) database.

**Fig 2:**
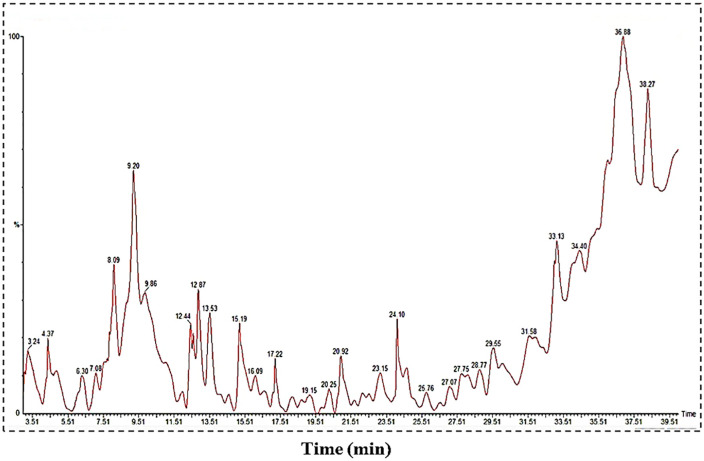
GC-MS Chromatogram of *Typha domingensis* plant extract.

In GC-MS analysis, 27 compounds were identified from *Typha domingensis*. Fig 2 represents the distinct GC-MS chromatogram. The bioactive compounds identified from *Typha domingensis* were represented by their retention time (RT), molecular formula, molecular weight and peak area (%) in Table 2.

**Table 2:** Phytochemical composition of *Typha domingensis* plant extract.

Serial No.	Retention Time (RT)	Compounds	Molecular Weight	Molecular Formula	% Area
1	3.24	2-ethylpiperidine	113	C_7_H_15_N	0.01
2	4.37	Pyrazine, methyl	94	C_5_H_6_N_2_	1.57
3	6.30	8-azabicyclo [5,2,0] nonan-9-one	139	C_8_H_13_ON	1.3
4	7.08	Cyclobutanone, 2-tetradecyl-	266	C_18_H_34_O	0.99
5	8.09	Dimexano	214	C_4_H_6_O_2_S_4_	6.64
6	9.20	Carbamic acid, butylmethyl-, methyl ester	145	C_7_H_15_O_2_N	24.76
7	9.86	3-pentenoic acid, 2,2-dimethyl-	128	C_7_H_12_O_2_	0.02
8	12.44	5h-5-methyl-6,7-dihydrocyclopentapyrazine	134	C_8_H_10_N_2_	0.40
9	12.58	Benzene, 1-chloro-2-methoxy-	142	C_7_H_7_OCl	2.05
10	12.87	Succinic acid, dodec-2-en-1-yl 2-naphthyl ester	410	C_26_H_34_O_4_	2.09
11	13.53	L-valine, n-(3-methylbut-2-enoyl)-, ethyl ester	227	C_12_H_21_O_3_N	1.98
12	14.60	2-pentenoic acid, ethyl ester	128	C_7_H_12_O_2_	0.25
13	15.19	1h-1,2,4-triazol-5-amine, 1-propyl-	126	C_5_H_10_ON_4_	3.13
14	16.09	4-fluorothiophenol	128	C_6_H_5_FS	1.08
15	17.22	4-acetoxy-3-methoxystyrene	192	C_11_H_12_O_3_	0.85
16	19.15	Dihydro-pseudosolasodine	415	C_27_H_45_O_2_N	0.82
17	20.25	6-hydroxybenzofuran-3-one	115	C_8_H_6_O_3_	0.75
18	20.92	Dl-proline, 5-oxo-, methyl ester	143	C_6_H_9_O_3_N	1.58
19	21.68	Dimethyl phthalate	194	C_10_H_10_O_4_	0.08
20	23.15	Phthalimide	147	C_8_H_5_O_2_N	1.51
21	24.10	4-methyl-2,5-dimethoxybenzaldehyde	180	C_10_H_12_O_3_	1.86
22	25.76	Tetradecanoic acid	228	C_14_H_28_O_2_	0.50
23	28.77	(E)-4-(3-hydroxyprop-1-en-1-yl)-2-methoxyphenol	180	C_10_H_12_O_3_	0.92
24	29.55	Cyclohexanecarboxylic acid, 4-hexyl-, 2,3-dicyano-4-ethoxyphenylest	382	C_23_H_30_O_3_N_2_	1.69
25	33.13	Cyclohexanecarboxylic acid, 4-hexyl-, 2,3-dicyano-4-ethoxyphenyl est	382	C_23_H_30_O_3_N_2_	3.03
26	36.88	Cholest-2-en-3-ol, 4-nitrobutanoate, (5. alpha.)-	501	C_31_H_51_O_4_N	23.19
27	38.27	Cholesteryl formate	414	C_28_H_46_O_2_	4.06

### Preparation of ZnO NPs

The following procedure, with a small modification, was used to synthesize the ZnO NPs**.** A certain quantity (5.0 g) of zinc nitrate hexahydrate $\left(Zn{\left(NO_{\text{3}}\right)}_{\text{2}}.\text{6}H_{\text{2}}O\right)$ salt was vigorously agitated in 100 mL of distilled water to dissolve it. 20 mL of aqueous *Typha domingensis* leaf extract was added to the solution, and it was vigorously stirred for two hours at 60 °C. In order to regulate the size of the ZnO nanoparticles, the pH of the mixture was adjusted to 10 using a 2.0 M NaOH solution.

**Fig 3:**
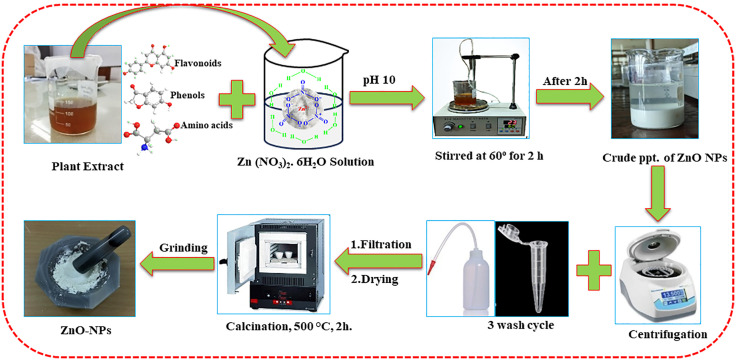
Diagrammatic depiction of the environmentally friendly synthesis of ZnO-NPs.

The solution turned white and formed a thick precipitate, which was then allowed to cool at room temperature. The mixture was then centrifuged for 10 minutes at 14,000 rpm and rinsed several times with ethanol and distilled water to eliminate any remaining impurities. After being dried for an entire night at 70 °C in an air oven, the product was calcined in a muffle furnace for two hours at 500 °C to obtain pure ZnO NPs [40]. The process is schematically represented in Fig 3.

### Proposed mechanism for the synthesis of ZnO NPs

**Fig 4:**
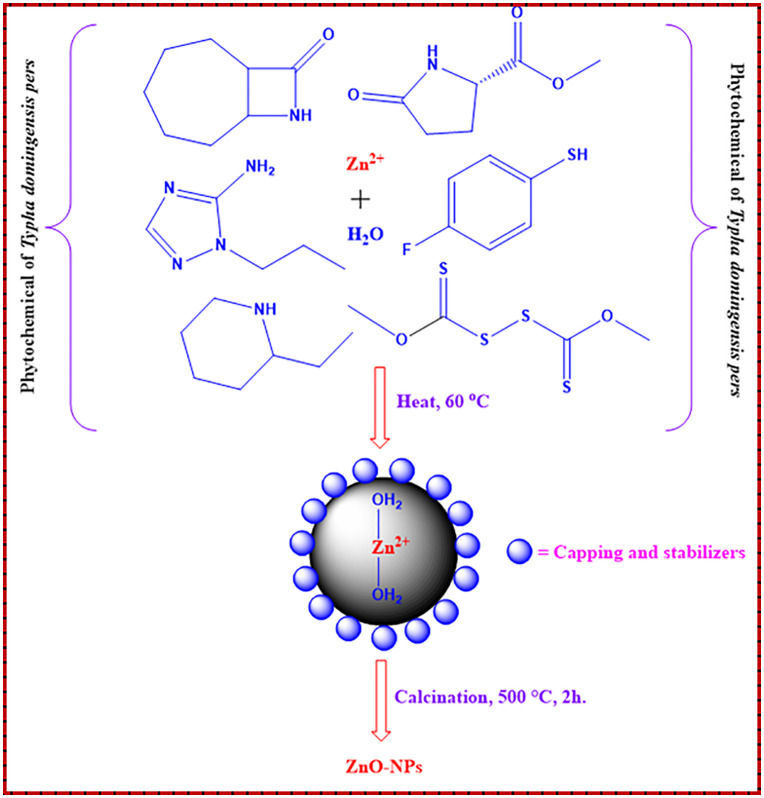
Proposed mechanism of ZnO NPs formation [41].

The ZnO NPs were synthesized using a plant extract. Constituents present in the extract acted as reducing, complexing, and stabilizing agents, facilitating the conversion of zinc ions into zinc hydroxide nanoclusters and subsequently into crystalline ZnO during thermal treatment. These constituents also adsorbed onto the nanoparticle surface, restricting particle growth and preventing agglomeration, which resulted in uniformly dispersed and stable ZnO NPs. The proposed mechanism for ZnO NPs formation is illustrated in Fig 4.

### Characterization of nanoparticles

The UV-Vis spectroscopic examination was performed using a SHIMADZU UV-1900i UV-Vis spectrophotometer (Tokyo, Japan). FTIR spectra were obtained on a SHIMADZU (Tokyo, Japan) IR Spirit Fourier transform infrared spectrophotometer using a KBr pellet and a scan rate of approximately 4 cm^−1^ s^−1^ at 25 °C. SEM measurements and EDX analysis were carried out on a Hitachi (Tokyo, Japan) SU-8000 microscope using accelerating voltages of 10 and 15 kV. The crystallographic solid-state structural study of the produced ZnO NPs was performed using X-ray diffraction measurements. The PANalytical X’Pert Pro (Japan) functioned at 45 kV and 50 mA, employing a 0.01° step scan across a 2θ range of roughly 10° to 80°, utilizing Cu-Kα radiation with a secondary monochromator at λ = 1.54 Å.

### Assessment of *in vitro* antioxidant properties of green synthesized ZnO NPs

ZnO NPs’ free radical scavenging ability was assessed utilizing the stable radical DPPH (2,2-Diphenyl-1-picrylhydrazyl). A specified volume of methanol was used to prepare 2 mL standard solutions of both ascorbic acid and commercial ZnO NPs at various concentrations (15.62, 31.25, 62.5, 125, 250 and 500 μg/mL). In addition, 2 mL solutions of ZnO NPs were also prepared at corresponding concentrations. Then, DPPH was weighed and dissolved in methanol to produce a 0.004% (w/v) solution. To ensure uniform dissolution, a sonicator was used. Then, 2 mL of freshly prepared DPPH solution was added to each test tube, and everything was properly vortexed. After that, the mixture was allowed to incubate at room temperature for 30 minutes in the dark. The UV-vis spectrophotometer was used to measure the absorbance at 516 nm. The percentage of inhibition, a measure of the free radical scavenging activity, was determined using the following formula [42–45].


$$\%\;of\;scavenging=\;\frac{\left(P_{c}-P_{s}\right)\times \text{1}\text{0}\text{0}}{p_{c}}$$ (1)

Where, $P_{c}$ denotes the absorbance of standard solutions, while $P_{s}$ represents the sample’s (ZnO NPs) absorbance at 516 nm.

### In vitro antifungal assay

The antifungal activity of biosynthesized ZnO NPs was tested against two plant pathogen strains, *Colletotrichum sp.* and *Fusarium sp.*, using the disc diffusion method on 5 mm discs. Stock solutions for the experiment were produced at concentrations of 1.0 mg/mL, 1.5 mg/mL, and 2.0 mg/mL. An electronic balance was used to precisely weigh ZnO NPs in amounts of 1.0 mg, 1.5 mg, and 2.0 mg in order to prepare the test samples. These amounts were diluted in 1 mL of dimethyl **s**ulfoxide (DMSO) in separate 1.5 mL Eppendorf tubes to make stock solutions at the specified concentrations. The solutions were mixed thoroughly using a vortex mixer and sonicated to ensure uniform dispersion of the nanoparticles in the solvent. For the spore suspension, spores were collected from four-day-old cultures grown at 28 °C on Potato Dextrose Agar (PDA) plates. The spores were then suspended in sterile distilled water containing 0.9% NaCl and three drops of Tween 80 to facilitate suspension. The mixture was standardized to a turbidity level of 0.5 McFarland using a densitometer. The suspension was mixed vigorously with a vortex for better dispersion. The resulting spore suspension was placed in a screw-cap tube, covered with foil for protection. When not in use, the McFarland standard was stored at room temperature (25 °C). Over time, the standard underwent precipitation and formed clumps. A spectrophotometer was used to adjust the fungal sample’s turbidity to a range of 0.08–0.12 at 625 nm by visually comparing it with the McFarland standard tube in well-lit conditions. After this adjustment, the suspension’s concentration was roughly 1 × 10^8 colony-forming units per milliliter (CFU/mL). A sterile cotton swab was dipped into the fungal suspension, which had a concentration of roughly 1 × 10^8 CFU/mL, for inoculation. The swab was gently pressed against the tube’s inner wall to remove any extra fungal debris. Next, the inoculum was uniformly applied to the PDA plates’ surface. In the antifungal screening, sample discs, standard discs, and blank discs were utilized. The PDA plates were divided into four sections: two sections served as replicates for the positive control (sample), one for the standard disc, and one for the blank disc (negative control). The stock solutions were applied to the discs using a micropipette until the discs were fully soaked. After the discs absorbed the nanoparticle solution, the control DMSO discs were removed, dried, and placed onto the agar media. To evaluate the antifungal activity of the ZnO NPs, the plates were incubated for 72 hours at 25 °C. Following this, the zones of inhibition were determined in millimeters using a slide caliper [46–49]. The following formula was used to determine the percentage of fungal growth inhibition [50].


$$Percentage\;of\;inhibition\;(\%)=\frac{D_{control}-D_{treatment}}{D_{control}}\times \text{1}\text{0}\text{0}\;$$ (2)

Here, $D_{control}$ represents the average mycelial growth in control petri plates, whereas $D_{treatment}$ represents the average mycelial growth in treated petri plates.

### In vitro Antibacterial Assay

The antibacterial activity of green-synthesized ZnO NPs was investigated against 2 gram-negative bacteria, *Coliform sp.* and *Salmonella sp.*, by employing the disk diffusion method on MacConkey Sorbitol Agar (SMAC) media. For this, the ZnO NPs were dissolved in a solvent in order to prepare a suspension with a concentration of 5.0 mg/mL. The bacterial inoculum was prepared using a fresh culture of *Coliform sp.* and adjusted to a 0.5 McFarland turbidity standard (about 1 × 10^8^ CFU/mL). Using a sterile swab, the bacterial suspension was equally distributed across the surface of the SMAC plates, and a sterile cork borer was used to make wells. After pipetting the ZnO NPs suspension (5000 ppm) into each well and incubating the plates for 24 hours at 37 °C, the antibacterial activity was calculated by measuring the inhibition zones surrounding the wells using a caliper [43,44].

### Statistical analysis

All experimental data are expressed as mean ± standard deviation (SD) based on triplicate measurements. Statistical analyses were conducted using IBM SPSS Statistics version 26.0, with a significance threshold set at p < 0.05. To assess the effects of antioxidant concentration and antioxidant type (synthesized ZnO NPs, commercial ZnO NPs, and ascorbic acid) on free radical inhibition activity, a two-way ANOVA was performed. The analysis evaluated the main effects of each factor as well as their interaction. Levene’s test confirmed the homogeneity of variances (p = 0.315), thereby meeting the assumptions for ANOVA. Effect sizes were reported using partial eta squared (η²), with values interpreted as small (0.01), moderate (0.06), or large (0.14). Additionally, a one-way ANOVA was employed to evaluate the antifungal activity of the samples by comparing inhibition zone diameters at different concentrations (1.0 mg/mL, 1.5 mg/mL, and 2.0 mg/mL) for each fungal strain. This analysis determined whether concentration had a statistically significant effect on fungal growth inhibition.

## Results and discussion

### UV-Visible spectroscopy

**Fig 5:**
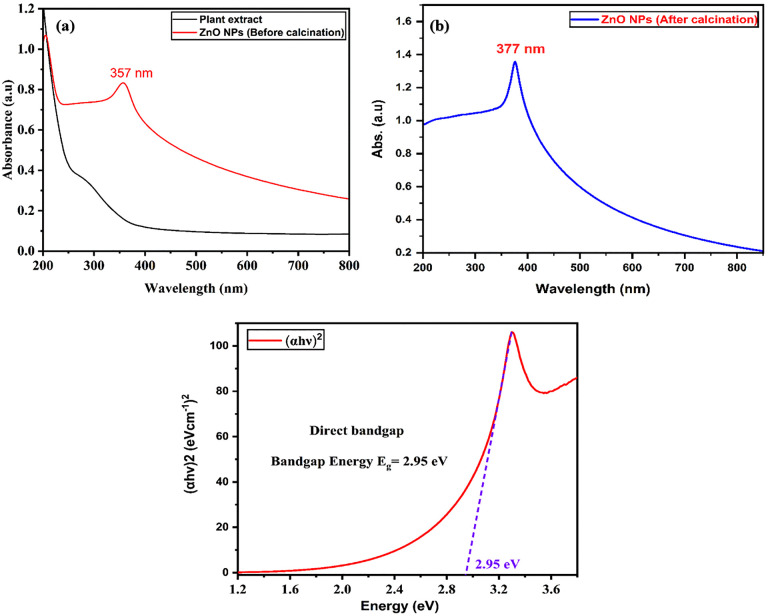
Optical characterization and bandgap estimation of green-synthesized ZnO nanoparticles. (a) UV-Vis spectra of plant extract and ZnO NPs (before calcination). (b) UV-Vis spectra of ZnO NPs (After calcination).

UV-Vis absorbance spectroscopy was used as a preliminary method for monitoring ZnO NPs formation in an aqueous solution [51,52]. The spectra of the plant extract, biosynthesized ZnO NPs before calcination, and after calcination are shown in Fig 5a and 5b. The plant extract exhibited a broad absorption band in the UV region without any sharp peaks, indicating the presence of various phytochemicals responsible for the reduction and stabilization of nanoparticles [53]. The ZnO NPs before calcination displayed a distinct absorption peak at 357 nm, which is characteristic of the intrinsic band-gap absorption of ZnO due to electron transitions from the valence band to the conduction band. After calcination, the absorption peak shifted to 377 nm, indicating a red shift that can be attributed to particle growth, improved crystallinity, and the removal of organic residues during thermal treatment. This shift also suggests a slight decrease in band-gap energy after calcination, consistent with the formation of well-defined crystalline ZnO nanoparticles. The absence of additional peaks in the spectrum also demonstrates the purity of the biosynthesized ZnO-NPs [54–57].

The observed absorption peak could be caused by either surface plasmon resonance (SPR), influenced by the quantum size effect [58,59] or semiconductor band gap transition [60,61]. A semiconductor’s band gap, which is the difference in energy between the valence band and the lowest conduction band, determines the amount of photon energy required to generate electrons and holes. To calculate the band gap energy of ZnO NPs, we analyzed the absorption spectra using Tauc’s equation (Equation 3) for direct band gap materials, as illustrated in Fig 5c [62].


$$(\alpha h\upsilon {)}^{\text{2}}=k\;\left(h\upsilon -E_{g}\right)\;\;$$ (3)

In this formula, the absorption coefficient is denoted by α; hυ, Eg and k represent the photon energy, the band gap energy and a constant. This could also be written as:


$$\;{\left(\text{2}.\text{3}\text{0}\text{3}\times A\times \text{1}\text{2}\text{4}\text{0}/\lambda \right)}^{\text{2}}=k\;\left(\frac{\text{1}\text{2}\text{4}\text{0}}{\lambda \;}-E_{g}\right)\;\;$$ (4)

In this case, λ is the wavelength obtained from the absorption spectra, and A is the absorbance. Equation 4 is plotted to create an absorption curve, and the tangent to this curve indicates the energy band gap of the nanoparticles. Prior studies have demonstrated that the band gap energies of biosynthesized ZnO NPs ranged from 2.88 to 3.10 eV [60]. Based on the results shown in Fig 5c, ZnO NPs’ band gap energy fell within the designated range. In this study, the band-gap energy for synthesized ZnO NPs was determined to be 2.95 eV [63].

### Fourier transform infrared spectroscopy (FT-IR)

**Fig 6:**
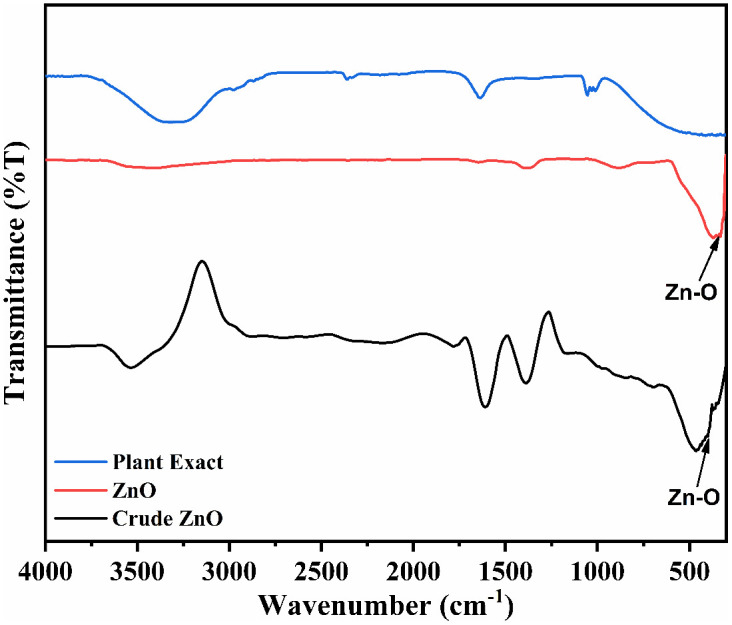
FT-IR spectra of plant extract, crude ZnO (before purification and calcination) and synthesized ZnO NPs (after purification and calcination).

The FTIR spectra of *Typha domingensis* extract, crude ZnO NPs, and calcined ZnO NPs are presented in Fig 6. The extract spectrum showed characteristic peaks at around 3401 cm ⁻ ¹ (O–H stretching), 2350 cm^-1^ (C–O stretching), 1645 cm ⁻ ¹ (C = O stretching of carbonyl groups), and a peak near 1050 cm ⁻ ¹ (C–O–C stretching), which are associated with phenols, flavonoids, tannins, and other phytochemicals present in the leaf extract [64–66]. In the ZnO NPs spectra, these peaks either shifted, decreased in intensity, or disappeared, indicating that these functional groups were involved in the reduction of Zn² ⁺ ions and in the stabilization of the formed nanoparticles. The strong absorption band around 440 cm ⁻ ¹, observed in both crude and calcined ZnO NPs, corresponds to Zn–O stretching vibrations, confirming ZnO formation. The disappearance of several organic peaks after calcination further suggests the removal of residual plant-derived organic matter, leaving behind pure ZnO NPs. These observations support the role of *T. domingensis* phytochemicals as both reducing and capping agents in the green synthesis process. A tabular summary of FTIR peak positions along with their corresponding functional group assignments for enhanced clarity and understanding of plant-derived capping agents (Table 3).

**Table 3:** FTIR spectra of *Typha domingenis* plant extract and calcined ZnO NPs.

Functional groups	Absorption bands in Plant extract (cm^-1^)	Absorption bands in calcined ZnO NPs (cm^-1^)
**O–H**	3401	3513 (shifted and decreased)
**C–O**	2350	Disappeared
**C = O**	1645	1645 (decreased)
**C–O–C**	1050	950 (shifted and decreased)
**Zn–O**	–	440

### Analysis of scanning electron microscopes (SEM)

**Fig 7:**
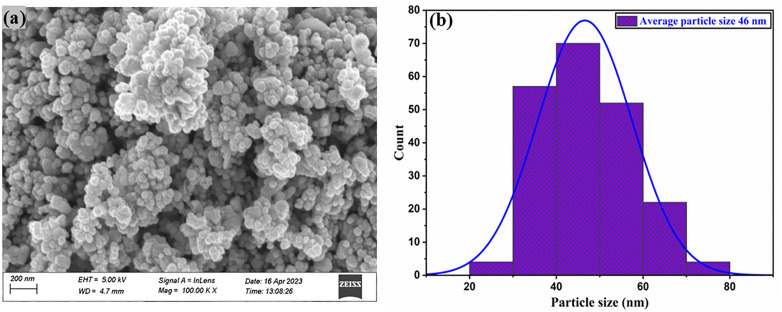
Morphological characterization of calcined ZnO nanoparticles. (a) SEM image and (b) particle size distribution of calcined ZnO NPs.

A scanning electron microscope (SEM) utilizes a high-energy electron beam to examine and capture images of samples [67,68]. The interaction between electrons and the atoms of the sample produces signals that convey details about the sample’s composition and surface characteristics [69,70]. SEM was used to determine the particle size and surface morphology of calcined ZnO NPs synthesized using green techniques. The SEM results revealed a predominantly spherical morphology, as illustrated in Fig 7a. A few particles agglomerated, most likely as a result of interactions between magnetic dipoles and the surface free energy of the nanoparticles. The data suggest that the particles are in a homogeneous state, which has a significant impact on their various activities.

The average size of the generated particles was approximately 46 nm; larger particles are linked to particle aggregation. The histograms in Fig 7b demonstrate the size distribution of ZnO NPs [71,72].

### Analysis of energy dispersive X-rays (EDX)

**Fig 8:**
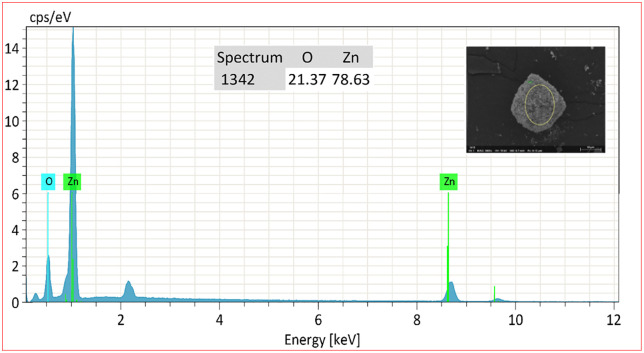
EDX Spectrum of calcined ZnO NPs.

The purity of the biosynthesized product was confirmed using EDX measurements. The EDX spectrum for ZnO NPs (after calcination) displays distinct peaks corresponding to the zinc and oxygen elements, indicating that the synthesized ZnO NPs are predominantly free of impurities, as illustrated in Fig 8. The EDX findings showed a significant emission energy at 1 keV, which aligns with the binding energy of zinc (78.63%), and at 0.5 keV for oxygen (21.37%), confirming the accurate identification of ZnO [69,73].

### X-ray diffraction (XRD) analysis of ZnO NPs

**Fig 9:**
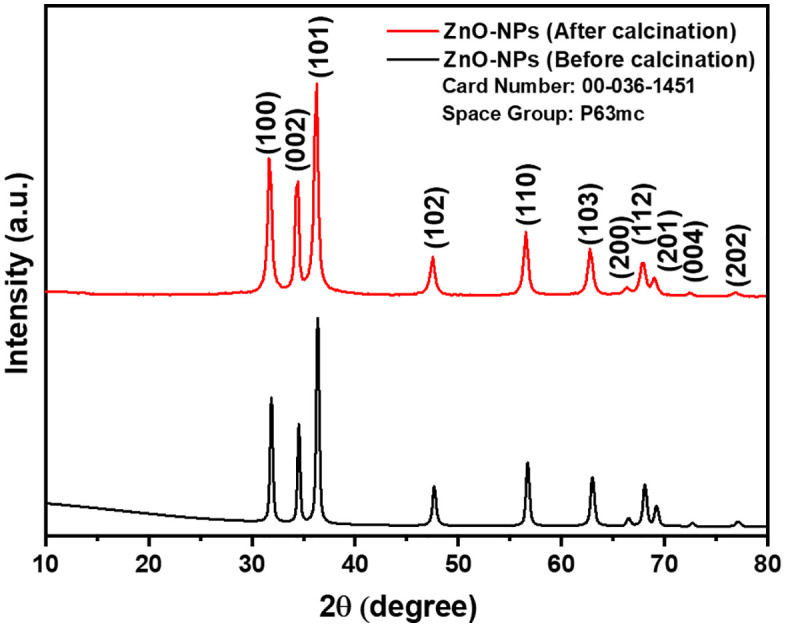
XRD Plot of ZnO NPs before and after calcination.

The XRD patterns of ZnO nanoparticles before and after calcination are shown in Fig 9. After calcination, the XRD pattern of ZnO NPs reveals diffraction peaks at 2θ angles of 31.86°, 34.52°, 36.35°, 47.64°, 56.69°, 62.94°, 66.46°, 68.04°, 69.17°, 72.62°, and 77.00°, which are associated with lattice planes (100), (002), (101), (102), (110), (103), (200), (112), (201), (004), and (202), respectively [74]. The observed peaks correspond to the reference peaks found in JCPDS card No: 00-036-1451, suggesting that the ZnO NPs possess a hexagonal wurtzite structure with a space group of p63mc [75–78]. The lattice parameters of ZnO NPs, such as unit cell volume, crystallite size, micro strain, and dislocation density, are presented in equations 5–9. The lattice parameter “a” for the synthesized ZnO NPs was estimated as 3.2504 Å (0.3250 nm), and possessed an exceptional degree of crystallinity as presented in Equation 5 [79]. Additionally, the volume of the unit cell for the green nanoparticles was determined to be 34.3408 Å³ (0.0343 nm³) by employing Equation 6 [80]. The average size of the crystallites in the ZnO nanoparticles was calculated using the Debye-Scherrer formula, as represented in Equation 7. In this equation, D denotes the size of the crystallites, λ refers to the wavelength of Cu Kα radiation at 1.5406 Å, β indicates the full-width at half maximum (in radians) of the peak, and θ indicates the angle of diffraction according to Bragg’s law (in degrees). The average size of the crystallites for green-synthesized ZnO-NPs was determined to be 26.74nm [81]. Another important factor to consider is the micro strain of NPs, which was determined using Equation 8.

The determined micro strain of the ZnO-NPs was 2.9727 × 10 ⁻ ³, suggesting the presence of a crystallographic defect, which points to irregularities in the crystal structure. [79]. The dislocation density of the ZnO nanoparticles was determined mathematically using the previously mentioned model Equation 9. The dislocation density of ZnO NPs was determined to be 1.4128 × 10 ⁻ ³, highlighting their electrochemical and catalytic characteristics [79]. Furthermore, the XRD pattern revealed no additional peaks beyond the distinctive ZnO peaks, confirming the purity of the synthesized ZnO-NPs [61,82–84].


$$\text{Lattice}\;\text{parameter},\;\text{a}\;(\text{nm})=\frac{\lambda }{\text{2}\;sin\theta }\sqrt{h^{\text{2}}+k^{\text{2}}+l^{\text{2}}}$$ (5)

$$\text{Unit}\;\text{volume}\;V\left({nm}^{\text{3}}\right)=a^{\text{3}}$$ (6)

$$\text{Average}\;\text{crystallite}\;\text{size}\;\text{D}\;(\text{nm})=\frac{\text{0}\mathrm{.9}\lambda \;}{\beta \;cos\theta }$$ (7)

$$\text{Micro}\;\text{strain}\;(\epsilon )\times {\text{10}}^{-\text{3}}=\frac{cos\theta }{\text{4}}$$ (8)

$$\text{Dislocation}\;\text{density}\;(\delta )\times {\text{10}}^{-\text{3}}\;\left(\text{lines}/{\text{nm}}^{\text{2}}\right)=\frac{\text{1}}{D^{\text{2}}}$$ (9)

**Fig 10:**
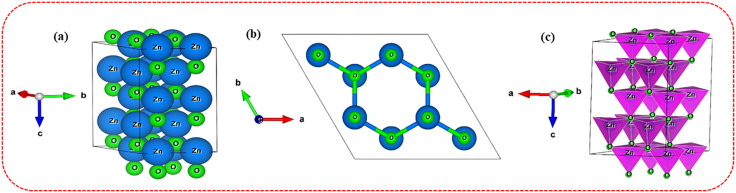
Crystal structure representations of ZnO nanoparticles. (a) Crystal structure of ZnO NPs in the single unit cell; (b) Crystal structure of ZnO-NPs in a single layer; (c) polyhedral structure of ZnO NPs.

In addition, the crystal structure was developed utilizing Vesta (3.90) software and XRD data, as illustrated in Fig 10(a-c). Now, the hexagonal wurtzite structure of zinc oxide is observable. Moreover, the unit cell structures of ZnO NPs consist of 64 atoms, 80 bonds, and 20 polyhedra [85].

### Antioxidant activity of ZnO NPs

**Fig 11:**
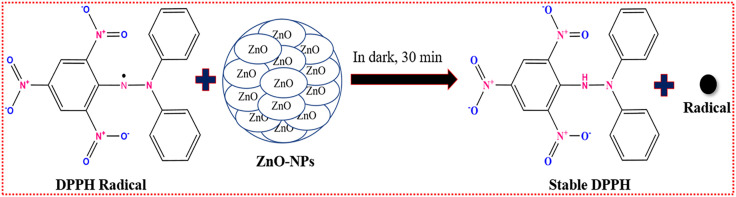
Schematic mechanism of DPPH scavenging by ZnO NPs.

All applications were carried out using calcined ZnO NPs. In biological systems, nanoparticles are becoming recognized for their high antioxidant properties, which play an important role in reducing oxidative stress, a fundamental contributor to cellular damage [44]. Oxidative stress arises when free radicals, which are reactive molecules possessing one or more unpaired electrons, are produced through the interactions of molecular oxygen with biomolecules. These free radicals have the potential to disrupt vital cellular components like lipids, proteins, and DNA, leading to significant cellular harm and ultimately impacting cell health [86,87]. ZnO NPs have the potential to mitigate these effects by neutralizing free radicals and shielding biological structures from oxidative damage. They can efficiently scavenge reactive species thanks to their antioxidant potential, which is derived from processes like electron donation and catalysis [88]. In this study, the capacity of ZnO NPs to scavenge free radicals was assessed using the stable radical DPPH (2,2-Diphenyl-1-picrylhydrazyl), offering important perspectives on their potential role in cellular protection [89]. DPPH is a stable nitrogen-centered free radical that is widely employed to scavenge resting radicals in compounds or plant extracts. The stable DPPH radical is reduced by gaining a hydrogen or an electron [69]. A possible mechanism of DPPH scavenging by calcined ZnO NPs is depicted in Fig 11. At a concentration of 500 µg/mL, ascorbic acid (control) showed a scavenging activity of 85.23 ± 3.26% (Table 3), while commercial ZnO NPs and synthesized ZnO NPs exhibited a scavenging activity of 78.09 ± 2.83% (Table 4) and 74.19 ± 2.93% (Table 5). Based on prior research, at an equivalent concentration of 1000 µg/mL, ZnO NPs synthesized from Fumaria parviflora extract exhibited a major antioxidant property, assessed using DPPH scavenging methods, yielding a percentage of 72.12% in comparison to ascorbic acid’s 94.25% [43]. Moreover, ZnO NPs derived from *Mollugo oppositifolia* leaf extract and *Trianthema portulacastrum* exhibited antioxidant activity using DPPH scavenging techniques with values of 93 ± 0.1% and 94 ± 0.2%, respectively, in contrast to ascorbic acid’s 96 ± 0.5% at the same 500 µg/mL concentration [90]. Furthermore, at a concentration of 300 µg/mL, ZnO NPs mediated by *Lentinula edodes (L. edoes)* exhibited the maximum scavenging activity 65.7 ± 1.09% in comparison to ascorbic acid 91.02 ± 0.54 [88].

**Table 4:** Percentage of inhibition and IC50 of ascorbic acid.

Concentration (µg/mL)	Absorbance of Control	Absorbance of Sample	% of inhibition	IC_50_ (µg/mL)
15.62	1.05	0.521	50.38 ± 2.02	16.66
31.25	1.05	0.426	59.00 ± 2.50	
62.50	1.05	0.375	64.28 ± 2.57	
125	1.05	0.295	71.90 ± 2.88	
250	1.05	0.217	79.33 ± 3.17	
500	1.05	0.155	85.23 ± 3.26	

**Table 5:** Percentage of inhibition and IC_50_ of Commercial ZnO NPs.

Concentration (µg/mL)	Absorbance of Control	Absorbance of Sample	% of inhibition	IC_50_ (µg/mL)
15.62	1.05	0.560	46.66 ± 1.50	28.45
31.25	1.05	0.499	52.47 ± 2.10	
62.50	1.05	0.385	63.33 ± 2.23	
125	1.05	0.316	69.90 ± 2.62	
250	1.05	0.282	73.14 ± 2.50	
500	1.05	0.230	78.09 ± 2.83	

**Fig 12:**
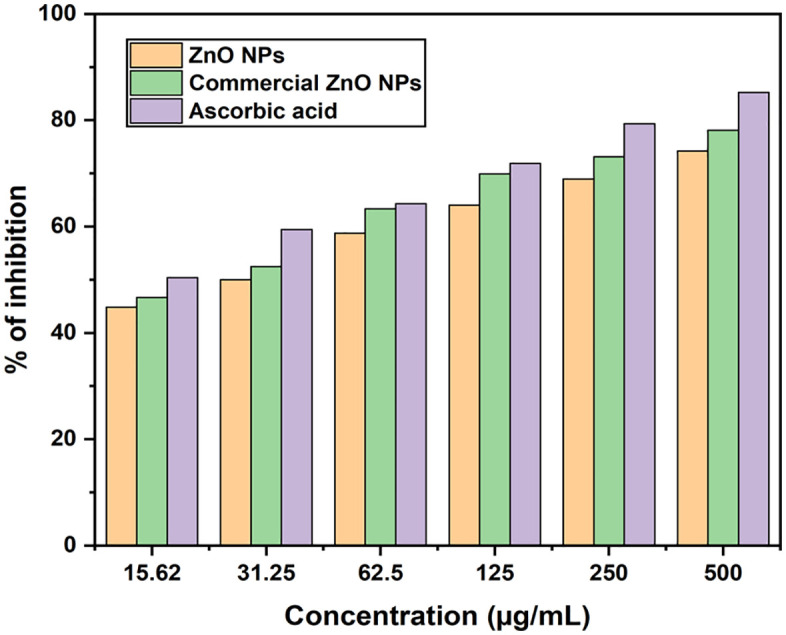
DPPH radical scavenging assay of ascorbic acid, commercial ZnO NPs and synthesized ZnO NPs.

The synthesized ZnO NPs exhibited an IC₅₀ value of 32.25 µg/mL (Table 6). In comparison, ascorbic acid demonstrated an IC₅₀ of 16.66 µg/mL, while commercial ZnO NPs showed an intermediate IC₅₀ value of 28.45 µg/mL. These results indicate that the synthesized ZnO NPs show their strong antioxidant efficiency. Moreover, a previous report [74], documented IC₅₀ value of 28.11 ± 0.01 µg/mL for ZnO NPs and 11.50 ± 0.03 µg/mL for ascorbic acid. The scavenging assay of ZnO nanoparticles was compared with ascorbic acid (standard) and commercial ZnO NPs, as shown in Fig 12. The scavenging activity of ZnO NPs increases as the sample concentration rises. This study shows that ZnO nanoparticles are highly effective at scavenging free radicals and can be a valuable source of antioxidants for antioxidant-based therapy.

**Table 6:** Percentage of inhibition and IC_50_ of synthesized ZnO NPs.

Concentration (µg/mL ^1^)	Absorbance of Control	Absorbance of Sample	% of inhibition	IC_50_ (µg/mL)
15.62	1.05	0.579	44.85 ± 1.72	32.25
31.25	1.05	0.525	50.00 ± 1.93	
62.50	1.05	0.433	58.76 ± 2.30	
125	1.05	0.378	64.00 ± 2.51	
250	1.05	0.326	68.95 ± 2.72	
500	1.05	0.271	74.19 ± 2.93	

A two-way ANOVA was conducted to evaluate the effects of antioxidant concentration, antioxidant type (green ZnO NPs, commercial ZnO NPs, and ascorbic acid), and their interaction on DPPH free radical inhibition activity. Levene’s test confirmed the assumption of homogeneity of variances (p = 0.315), validating the appropriateness of the analysis.

As shown in Table 7, the results indicate statistically significant main effects for both antioxidant concentration (F (5, 36) = 97.86, p < 0.001, η² = 0.94) and antioxidant type (F (2, 36) = 30.12, p = 0.001, η² = 0.52). This confirms that both the dosage and the source of the antioxidant significantly influence free radical inhibition. Moreover, a significant interaction effect between concentration and type (F (10, 36) = 5.08, p = 0.009, η² = 0.29) suggests that the effectiveness of each antioxidant varies depending on the concentration used. The interaction effect supports that the difference between antioxidant types becomes more pronounced at higher concentrations, with green ZnO NPs exhibiting superior scavenging capability. These findings reinforce the dose-dependent and formulation-dependent nature of ZnO NPs’ antioxidant performance and highlight the advantage of green synthesis in enhancing their biological efficacy.

**Table 7:** Tests of between-subjects effects.

Source	F	Sig.	Partial Eta Squared
Concentration	97.86	0.000	0.94 (very strong effect)
Type	30.12	0.001	0.52 (strong effect)
Concentration × Type	5.08	0.009	0.29 (moderate interaction)

### Antimicrobial activities

#### In vitro antifungal properties of ZnO NPs.

**Fig 13:**
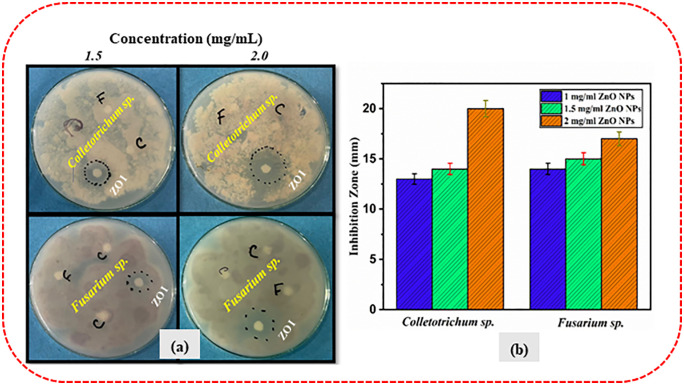
*In vitro* antifungal performance of green-synthesized ZnO nanoparticles against plant pathogenic fungi. (a) Antifungal activities of ZnO NPs against two fungal strains: *Colletotrichum sp.* and *Fusarium sp.* at two different concentrations (1.5 and 2.0 mg/mL). (b) Measurement of the inhibition zone of ZnO nanoparticles against two fungal species (*Colletotrichum sp.* and *Fusarium sp.*) at three different concentrations (1.0, 1.5 and 2.0 mg/mL).

The in vitro antifungal test revealed that ZnO NPs produced from *Typha domingensis* displayed considerable antifungal effectiveness against *Colletotrichum sp.* and *Fusarium sp.* in a dose-dependent manner, as indicated by the ZOI recorded (Table 8) after 72 hours of incubation. The ZOI increased consistently with nanoparticle concentration, indicating that higher concentrations of ZnO NPs lead to greater antifungal efficacy against all the fungal pathogens, as illustrated in Fig 13a and 13b. For *Colletotrichum sp*., a concentration of 1.0 mg/mL resulted in a 13 ± 0.52 mm inhibition zone with 14.44% inhibition, while 1.5 mg/mL produced a 14 ± 0.56 mm zone with 15.55% inhibition. The highest activity was observed at 2.0 mg/mL, with a 20 ± 0.80 mm inhibition zone and 22.22% inhibition. Similarly, for *Fusarium* sp., the inhibition zone at 1.0 mg/mL was 14 ± 0.56 mm with 15.55% inhibition, increasing to 15 ± 0.60 mm and 16.66% at 1.5 mg/mL. At a concentration of 2.0 mg/mL, the highest level of inhibition was observed, resulting in an inhibition zone measuring 17 ± 0.68 mm and an inhibition percentage of 18.89%. Notably, *Colletotrichum sp.* showed larger inhibition zones (20 ± 0.80 mm) compared to *Fusarium sp.* (17 ± 0.68 mm) at the same concentration (2.0 mg/mL), suggesting species-specific variations in susceptibility, possibly due to differences in cell wall composition, metabolism, or resistance mechanisms. With the highest concentration (2.0 mg/mL) exhibiting the most significant inhibition against both fungus species, these findings reveal a dose-dependent rise in antifungal activity. The observed antifungal activity may be attributed to their special physicochemical characteristics, such as their tiny size and wide surface area, which improve their interaction with fungal cells.

**Table 8:** The assessment of the inhibition zone of biosynthesized ZnO NPs at varying concentrations (1.0, 1.5 and 2.0 mg/mL) against two fungal strains, *Colletotrichum sp.* and *Fusarium sp.*

Fungal strain	Inhibition Zone(mm)		
**1.0 mg/mL**	**1.5 mg/mL**	**2.0 mg/mL**
*Colletotrichum sp.*	13 ± 0.52	14 ± 0.56	20 ± 0.80
*Fusarium sp.*	14 ± 0.56	15 ± 0.60	17 ± 0.68

One-way ANOVA was performed to evaluate the effect of different concentrations (1.0 mg/mL, 1.5 mg/mL, and 2.0 mg/mL) on the inhibition zone of fungal growth. The results revealed that the differences in inhibition zones among concentrations were statistically significant for both fungal strains. The p-value for *Colletotrichum sp.* was 0.00001, and for *Fusarium sp.* was 0.00127, indicating a significant concentration-dependent antifungal activity (p < 0.05**).** These results indicate that increasing the concentration of the antifungal agent significantly enhances its inhibitory effect on both fungal strains.

#### In vitro antibacterial properties of ZnO NPs.

**Fig 14:**
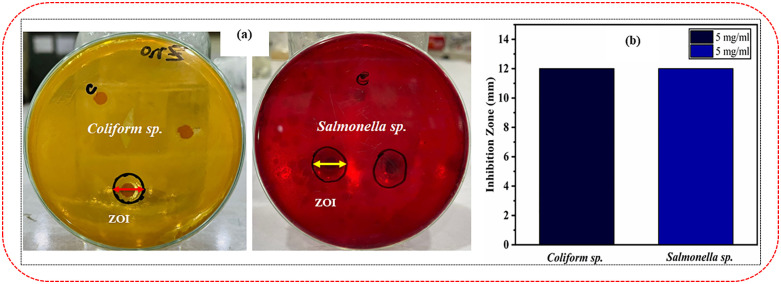
Antibacterial activity of green-synthesized ZnO nanoparticles against gram-negative bacteria. (a) Antibacterial activities of ZnO NPs against two gram*-*negative bacteria *(Coliform sp.* and *Salmonella sp.)* at a concentration of 5.0 mg/mL. (b) Measurement of the inhibition zone of ZnO nanoparticles against two bacterial species *(Coliform sp.* and *Salmonella sp.)* at a concentration of 5.0 mg/mL, both showing a 12 mm ZOI (zone of inhibition).

The green-synthesized ZnO NPs demonstrated significant antibacterial potential against two gram-negative bacteria, *Coliform sp.* and *Salmonella sp.* Both showed a 13.33% inhibition (Fig 14a and 14b) in the disc diffusion assay conducted on MacConkey Sorbitol Agar (SMAC) at a 5.0 mg/mL concentration, indicating their potential antibacterial properties. Additionally, the use of green synthesis methods, involving bioactive compounds from plant extracts, may enhance the antimicrobial efficacy of the nanoparticles through synergistic effects. The selective use of SMAC as the growth medium ensured the targeted assessment of *Coliform sp.* and *Salmonella sp.*, making the results particularly relevant for applications in water and food safety, where these bacteria are common contaminants. Numerous investigations have demonstrated that ZnO NPs have strong antibacterial properties against both gram-positive and gram-negative microorganisms [43,44,65].

**Fig 15:**
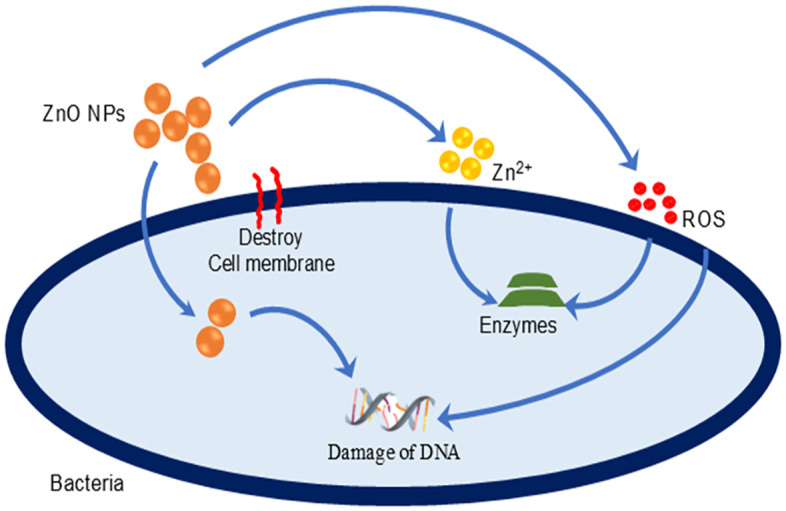
The plausible antibacterial mechanism of ZnO NPs [13].

Possible mechanisms of the antibacterial activity of ZnO NPs are also shown in Fig 15. ZnO NPs emit Zn^2+^ ions that are absorbed by bacterial cells. These ions can interfere with the cell’s enzymatic systems, thereby impeding normal cellular function. Reactive Oxygen Species (ROS) produced by ZnO NPs damage bacterial cells by disrupting critical biological components such as proteins, lipids, and DNA. Bacterial cells can either absorb ZnO NPs or engage directly with their surfaces. This interaction may damage the cell membrane, thereby compromising the cell’s integrity and potentially leading to its destruction [91,92].

## Conclusions

In conclusion, compared to traditional approaches, this study demonstrates a simple, economical, stable, sustainable, safe, and eco-friendly method for biosynthesizing ZnO NPs using *Typha domingensis* leaf extract. The phytochemicals in the extract acted as natural reducing and capping agents, as confirmed by GC–MS analysis. The characteristic UV–Vis absorption peak at 377 nm indicated ZnO NP formation, while FTIR analysis confirmed Zn–O bonding along with C–O and O–H functional groups. XRD analysis revealed a pure hexagonal wurtzite structure (space group P63mc) with an average crystallite size of ~26.75 nm calculated using Debye–Scherrer’s formula. SEM images showed predominantly spherical nanoparticles with some aggregation due to high surface energy, and EDX confirmed high purity. The biosynthesized ZnO NPs exhibited strong antioxidant activity (IC₅₀ = 32.25 µg/mL) and notable antimicrobial effects against selected pathogens, supporting potential applications in agriculture, biomedical products, and active food packaging.

### Limitations and future work

However, this study has certain limitations, including the absence of MIC/MBC determinations, a lack of gram-positive bacterial testing, and a limited range of pathogens examined. Future work should address these gaps by expanding antimicrobial screening, performing in vivo biocompatibility and toxicity studies, and exploring the incorporation of these nanoparticles into antimicrobial coatings, delivery systems, and scalable production processes.
